# Diagnostic and prognostic implications of parafibromin immunohistochemistry in parathyroid carcinomaT

**DOI:** 10.1042/BSR20181778

**Published:** 2019-04-30

**Authors:** Jung-Soo Pyo, Won Jin Cho

**Affiliations:** 1 Department of Pathology, Eulji University Hospital, Eulji University School of Medicine, 95 Dunsansero, Seo-gu, Daejeon 35233, Republic of Korea; 2 Department of Urology, Chosun University Hospital, Chosun University School of Medicine, 365 Pilmundaero, Dong-gu, Gwangju 61453, Republic of Korea

**Keywords:** diagnostic accuracy, immunohistochemistry, parathyroid carcinoma, parafibromin, prognosis

## Abstract

The aim of the present study was to elucidate the diagnostic and prognostic implications of parafibromin immunohistochemistry (IHC) in parathyroid carcinoma (PC). We performed a meta-analysis to examine the rate of loss of parafibromin expression from 18 eligible studies. In addition, a diagnostic test accuracy review was conducted to investigate the diagnostic role of parafibromin in PC. The rates of loss of parafibromin expression were 0.522 (95% CI: 0.444–0.599), 0.291 (95% CI: 0.207–0.391), 0.027 (95% CI: 0.011–0.064), and 0.032 (95% CI: 0.008–0.119) in PC, atypical parathyroid adenoma (APA), parathyroid adenoma (PA), and parathyroid hyperplasia, respectively. In the diagnostic test accuracy review for diagnosis of PC, the pooled sensitivity and specificity of parafibromin IHC was 0.53 (95% CI: 0.46–0.59) and 0.96 (95% CI: 0.95–0.97), respectively. The diagnostic odds ratio and the area under curve on summary receiver operating characteristic curve was 25.31 (95% CI: 8.91–71.87) and 0.7954, respectively. In addition, the meta-analysis demonstrated that loss of parafibromin expression was significantly correlated with worse disease-free survival (hazard ratio: 2.832; 95% CI: 1.081–7.421). Loss of parafibromin IHC expression was significantly higher in PC than in APA, PA, and parathyroid hyperplasia. Parafibromin IHC could be useful for diagnosis and prediction of prognosis of PC in daily practice.

## Introduction

Parathyroid carcinoma (PC) is a rare tumor amongst parathyroid tumors, which represents less than 1% of all cases [1]. Histologic diagnostic criteria for PC include invasion into surrounding tissues, capsular invasion, perineural invasion, and vascular invasion [1–3]. Cytological features, such as nuclear monotony, nucleoli, abnormal mitoses, are helpful for differentiation of PC from other parathyroid lesions [1–3]. In addition, histologically documented lymph node metastasis or distant metastasis is an important feature in diagnosis of malignancy [1,2]. Atypical parathyroid adenoma (APA) is a tumor with atypical cytological and architectural features, which does not fulfill the criteria for PC. Although the preoperative diagnosis of PC is required for a proper therapeutic plan, obtaining conclusive information from preoperative fine-needle aspiration or core needle biopsy is not recommended. In daily practice, fine-needle aspiration is not suitable method for differentiation between a benign and malignant tumor. In postoperative histological examination, it may be difficult to differentiate between PC and APA.

Previous studies introduced several immunohistochemical (IHC) biomarkers for PC including parafibromin, APC, galectin-3, PGP9.5, Ki67, and cyclin D1 [2,3]. Parafibromin is a protein product of the *CDC73/HRPT2* gene, which is associated with the hereditary hyperparathyroidism-jaw tumor syndrome and sporadic PCs [3]. The loss of parafibromin expression due to *CDC73/HRPT2* gene mutation correlates with cell proliferation, transcription, and histone modification [2,3]. However, the usefulness of these IHC markers in PC is unclear.

In the current study, we investigated the loss of parafibromin IHC expression in PC and compared this with various parathyroid lesions. A diagnostic test accuracy review assessed the role of parafibromin IHC in diagnosis of PC. In addition, a meta-analysis was performed to define the prognostic role of parafibromin IHC in PC.

## Materials and methods

### Published study search and selection criteria

Relevant articles were identified by a search of the PubMed and MEDLINE databases through 30 June 2018 using the key words ‘parathyroid’ and ‘parafibromin.’ The titles and abstracts of the articles were screened for exclusion. Review articles were further screened to find additional eligible studies. Searched results were then reviewed and included if (1) the study was performed in human parathyroid tissue and (2) there was information about the parafibromin IHC expression in various parathyroid lesions, and excluded if the articles were (3) case reports or non-original articles or (4) non-English language publications. The present study performed by Preferred Reporting Items for Systematic Reviews and Meta-Analyses (PRISMA).

### Data extraction

Data from all eligible studies were extracted by two independent authors. The included data were extracted from each of the eligible studies [2–19]: the first author’s name, year of publication, study location, antibody clone, and manufacturer, antibody dilution ratio and cut-off value, and number of patients analyzed. For meta-analysis, we extracted all data associated with IHC results. In addition, for quantitative aggregation of survival results, the correlation between parafibromin expression and survival rate was analyzed according to the hazard ratio using extraction of survival rates at specified times from survival curves [20]. The published survival curves were read independently by two authors in order to reduce reading variability. The hazard ratios (HRs) were then combined into an overall HR using Peto’s method [21].

### Statistical analysis

To perform the meta-analysis, data were analyzed by the Comprehensive Meta-Analysis software package (Biostat, Englewood, NJ, U.S.A.). The rates of loss of parafibromin IHC expression were investigated in various parathyroid lesions for the meta-analysis. For subgroup analysis, cut-off value 0% and >0% subgroups were subdivided according to the cut-off value for loss of parafibromin IHC expression. Heterogeneity between studies was checked using the Q and I^2^ statistics and presented using *P*-values. Additionally, a sensitivity analysis was conducted to assess the heterogeneity of eligible studies and the impact of each study on the combined effect. The meta-regression test was performed to elucidate the heterogeneity between two subgroups. For assessment of publication bias, Begg’s funnel plot and Egger’s test were performed. When a significant publication bias was found, the fail-safe N and trim-fill tests were additionally conducted to confirm the degree of publication bias. The results were considered statistically significant when *P*<0.05. Moreover, diagnostic test accuracy review was analyzed using the Meta-Disc program (version 1.4, unit of Clinical Biostatics, the Ramon y Cajal Hospital, Madrid, Spain) [22]. The summary receiver operating characteristic (SROC) curve was initially constructed by plotting ‘sensitivity’, and ‘1-specificity’ of each study and the curve fitting was performed through linear regression using the Littenberg and Moses linear model [23]. Because heterogeneity by evaluation criteria was present, the accuracy data were pooled by fitting a SROC curve and measuring the value of the area under the curve (AUC) [19]. An AUC close to 1 was a perfect test and an AUC close to 0.5 was considered as poor tests. In addition, the diagnostic odds ratio was calculated by the Meta-Disc program.

## Results

### Selection and characteristics of studies

**Figure 1:**
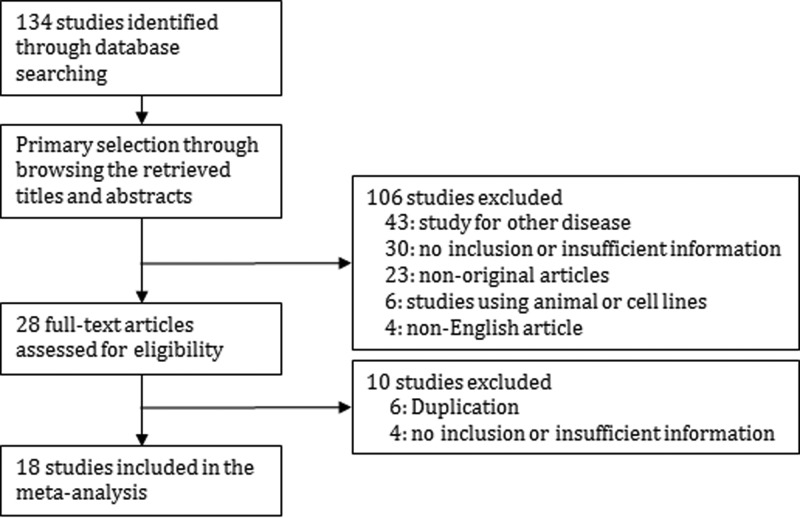
Flow chart of the study search and selection methods

A total of 134 reports were identified in the database search. A total of 34 were excluded due to lack of or insufficient information for parafibromin IHC expression. In addition, 82 reports focussed on other diseases (*n*=43), non-original articles (*n*=23), duplicated reports (*n*=6), studies using animal or cell lines (*n*=6), or articles in a language other than English (*n*=4) and were excluded. A total of 18 eligible studies were ultimately included. The current meta-analysis comprised 2123 parathyroid lesions, including 327 PCs (Figure 1 and Table 1).

**Table 1:** Main characteristics of the eligible studies

Study	Location	Corporation of antibody	Clone	Dilution	Cut-off value	Number of patients			
						PC	Atypical PA	PA	Hyperplasia
Cetani 2007	Italy	ND	511U	1:300	ND	11	4	22	-
Cetani 2013	Italy	Santa Cruz	ND	1:50	5%	34	-	-	-
Fernandez-Ranvier 2009	U.S.A.	Santa Cruz	2H1	1:100	0%	16	2	18	14
Guarnieri 2012	Italy	Santa Cruz	ND	1:200	0%	12	13	17	-
Hosny Mohammed 2017	Egypt	Santa Cruz	2H1	1:40	*	21	3	73	-
Howell 2009	Sweden	Santa Cruz	ND	ND	0%	9	-	78	16
Juhlin 2011	Sweden	Santa Cruz	2H1	ND	10%	22	11	43	-
Karaarslan 2015	Turkey	Santa Cruz	ND	1:100	0%	2	6	84	-
Kim 2012	Korea	Santa Cruz	2H1	1:50	1%	8	18	-	-
Kruijff 2014	Australia	ND	ND	ND	0%	27	54	-	-
Kumari 2016	India	Santa Cruz	ND	1:20	10%	14	19	194	-
Ozolins 2015	Germany	Abcam	ND	1:500	0%	8	10	964	-
Quinn 2015	U.S.A.	ND	ND	ND	ND	18	34	-	-
Selvan 2013	India	Santa Cruz	2H1	1:350	20%	5	-	-	-
Tan 2004	Singapore	ND	ND	ND	0%	58	-	48	25
Truran 2014	UK	Santa Cruz	2H1	1:150	0%	24	-	-	-
Wang 2012	China	Santa Cruz	2H1	1:40	0%	15	-	18	8
Witteveen 2011	U.S.A.	ND	ND	ND	0%	23	-	-	-

ND, No description.

* Using the outcome of multiplying the percentage of tumor cells stained (0–100) by staining intensity (0–3).

### Loss of parafibromin in parathyroid lesions

The estimated rate of loss of parafibromin IHC expression of PC was 0.522 (95% CI: 0.444–0.599) and was significantly higher than those of APA (0.291, 95% CI: 0.207–0.391), PA (0.027, 95% CI: 0.011–0.064), and parathyroid hyperplasia (0.032, 95% CI: 0.008–0.119) (Table 2). In subgroup analysis based on cut-off value, the estimated rates of cut-off 0% subgroup were lower than that of cut-off >0% subgroup. However, there was no significance difference of rates between cut-off 0% and >0% subgroups in PC (*P*=0.639) and atypical PA groups in the meta-regression test (*P*=0.766). In sensitivity analysis, no single study had a significant effect on the pooled estimates. No significant publication bias was observed in the primary tests (Begg’s funnel plot and Egger’s test) or in the secondary tests (fail-safe N and trim-fill tests).

**Table 2:** The rate of loss of parafibromin expression in parathyroid lesions

	Number of studies	Fixed effect (95% CI)	Heterogeneity (*P*-value)	Random effect (95% CI)	Egger’s test
Parathyroid carcinoma	18	0.508 (0.451–0.565)	0.062	0.522 (0.444, 0.599)	0.025
Non-Asia	12	0.503 (0.429–0.578)	0.020	0.519 (0.403–0.632)	0.147
Asia	6	0.514 (0.426–0.601)	0.527	0.514 [0.426–0.601)	0.008
Cut-off value 0%	10	0.498 (0.425–0.570)	0.169	0.506 (0.411–0.600)	0.248
Cut-off value >0%	6	0.533 (0.433–0.631)	0.159	0.548 (0.414–0.676)	0.263
Atypical parathyroid adenoma	10	0.308 (0.237–0.390)	0.276	0.291 (0.207–0.391)	0.076
Non-Asia	8	0.235 (0.151–0.348)	0.368	0.234 (0.143–0.358)	0.381
Asia	2	0.371 (0.268–0.487)	0.571	0.371 (0.268–0.487)	-
Cut-off value 0%	5	0.320 (0.223–0.434)	0.134	0.202 (0.081–0.419)	0.019
Cut-off value >0%	3	0.349 (0.206–0.526)	0.500	0.349 (0.206–0.526)	0.588
Parathyroid adenoma	12	0.067 (0.047–0.096)	0.001	0.027 (0.011–0.064)	0.003
Non-Asia	8	0.031 (0.015–0.063)	0.003	0.016 (0.004–0.061)	0.006
Asia	4	0.088 (0.058–0.131)	0.365	0.083 (0.049–0.136)	0.106
Cut-off value 0%	7	0.036 (0.016–0.078)	0.002	0.016 (0.003–0.076)	0.008
Cut-off value >0%	4	0.082 (0.055–0.123)	0.097	0.044 (0.014–0.132)	0.089
Parathyroid hyperplasia*	4	0.032 (0.008–0.119)	0.961	0.032 (0.008–0.119)	0.015
Non-Asia	2	0.031 (0.004–0.191)	0.949	0.031 (0.004–0.191)	-
Asia	2	0.033 (0.005–0.199)	0.590	0.033 (0.005–0.199)	-

* All criteria 0%.

### Diagnostic test accuracy review of parafibromin IHC in PC

**Figure 2:**
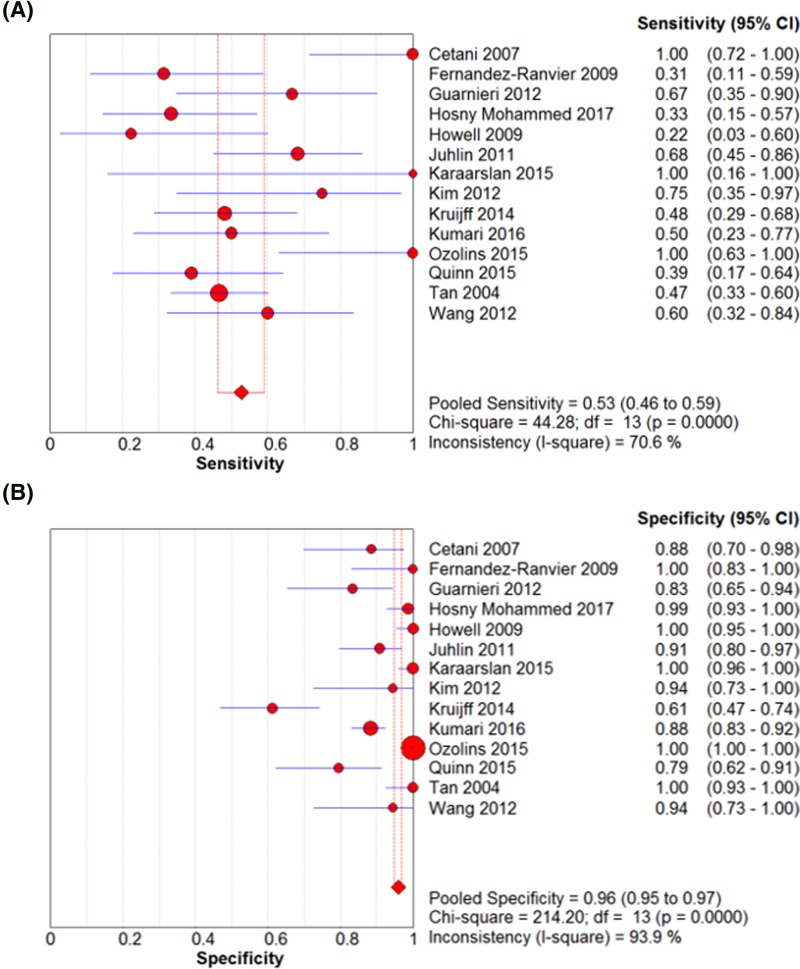
The forest plots for the sensitivity and specificity. The pooled sensitivity (**A**) and specificity (**B**) of parafibromin IHC in PC.

**Figure 3:**
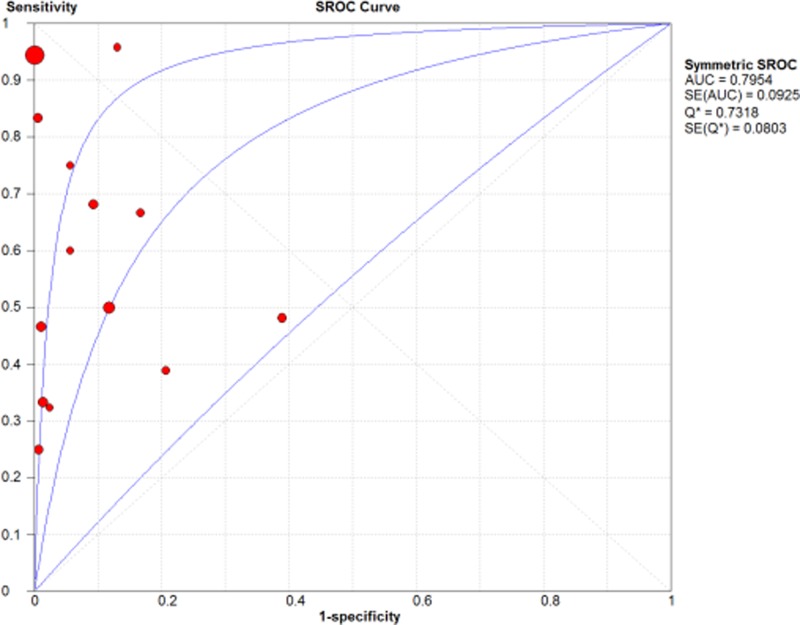
SROC curve of parafibromin IHC

A diagnostic test accuracy review was conducted to elucidate the role of parafibromin IHC in diagnosis of PC. The pooled sensitivity and specificity were 0.53 (95% CI: 0.46–0.59) and 0.96 (95% CI: 0.95–0.97), respectively (Figure 2). The ranges of sensitivity and specificity of eligible studies were 0.22–1.00 and 0.61–1.00, respectively. The pooled diagnostic OR was significantly high at 25.31 (95% CI: 8.91–71.87). The value of AUC on the SROC curve was 0.7954 (Figure 3). To evaluate the optimal cutoff, subgroup analysis was performed based on cut-off values. In cutoff 0% subgroup, the pooled sensitivity and specificity, diagnostic OR, and the value of AUC were 0.50 (95% CI: 0.42–0.59), 0.98 (95% CI: 0.97–0.99), 48.20 (95% CI: 7.02–333.15), and 0.6617, respectively. In the cutoff >0% subgroup, the pooled sensitivity and specificity, diagnostic OR, and the value of AUC were 0.54 (95% CI: 0.41–0.66), 0.91 (95% CI: 0.88–0.94), 15.93 (95% CI: 7.05–36.01), and 0.8339, respectively.

### Correlation between parafibromin IHC and survival in PC

**Figure 4:**
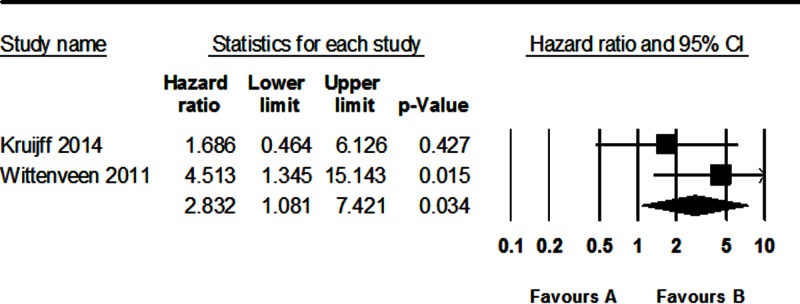
Forest plot diagram for the correlation between loss of parafibromin IHC and disease-free survival in PC

To elucidate the prognostic implication of parafibromin IHC, a meta-analysis was performed. In PC patients, loss of parafibromin expression was significantly correlated with worse disease-free survival (HR: 2.832; 95% CI: 1.081–7.421; Figure 4). However, the correlation between parafibromin IHC and overall survival could not be investigated because only one study had the information.

## Discussion

Although the sensitivity and specificity of parafibromin IHC for diagnosis of PC has been introduced and studied [2], the diagnostic and prognostic implication in PC is not fully elucidated. The current study is the first meta-analysis of published studies of the diagnostic and prognostic roles of parafibromin IHC in PC.

Cytological and architectural features suggesting PC include a thick capsule with fibrous septa dividing the gland, nuclear monotony, nucleoli, abnormal mitoses, invasion into surrounding tissues, capsular invasion, perineural invasion, and vascular invasion [1–3]. In addition, histologically documented lymph node metastasis or distant metastasis is an important feature in diagnosis of malignancy [1–3]. In a previous study, some parathyroid tumors diagnosed as benign showed recurrence or were metastatic on follow-up [24]. In addition, aggressive behavior was identified in only 15% of histologically diagnosed PC [25]. However, it is not easy to differentiate between PC and APA in daily practice through histology of primary tumor. So, ancillary tests, such as IHC, can be helpful for diagnosis and differentiation of PC and APA.

In the current meta-analysis, the rate of loss of parafibromin IHC expression was significantly higher than that of APA, PA, and parathyroid hyperplasia (Table 2). Loss of parafibromin IHC expression could be useful for diagnosis of PC as ancillary test. The pooled estimated specificity was 0.95 (95% CI: 0.93–0.96). However, Quinn et al. reported that there was no significant difference between PC and APA (38.0 vs 21.0%, *P*=0.342) [17]. Unlike other IHC markers for PC, parafibromin is a negative marker and the loss of parafibromin IHC expression is correlated with PC. In assessment of the loss of parafibromin IHC expression, the cut-off value may be important to decide the positivity. In our meta-analysis, the rates of loss of parafibromin IHC expression varied according to the cut-off value. When the cut-off value was high, the rate of loss of parafibromin IHC expression was high (Table 2). However, in diagnostic test accuracy review, the sensitivity of cutoff 0% subgroup was higher than that of cutoff >0% subgroup (0.74 vs 0.47).

Diagnosis of PC is confirmed through postoperative pathologic examination. When parathyroid lesion is diagnosed as PC, reoperation, such as thyroidectomy or neck dissection, will be needed. In this situation, information via preoperative examination can be useful to decide the therapeutic plan. However, preoperative fine-needle aspiration or core needle biopsy cannot be helpful in parathyroid lesions because these preoperative examinations are limited to differentiate between benign and malignancy [26,27]. In addition, such as fine-needle aspiration or core needle biopsy, the application of combination of several markers could be impossible from limited specimen. Therefore, it can be important to find and evaluate the most effective marker, but not combination of markers. In preoperative fine-needle aspiration or core needle biopsy, the parafibromin IHC expression can be useful for assessment of aggressive parathyroid lesion using minimal ancillary test.

As previously described, APA do not fulfill the cytological and architectural criteria for PC. In the current meta-analysis, the loss of parafibromin IHC expression was identified in 29.1% of APA. This rate was significantly lower than that of PC and significantly higher than that of PA. In the previous study, about 10% of APA with loss of parafibromin expression was recurred [13]. However, in APA with parafibromin expression, tumor recurrence was not found. The prognosis and tumor behavior of APA with loss of parafibromin expression could be differed with PA without atypism and APA with parafibromin expression. In the previous studies, loss of parafibromin IHC expression was significantly correlated with worse survival rate [5]. In the present study, loss of parafibromin expression was significantly correlated with worse disease-free survival in PC. Loss of parafibromin expression may have predictive role for parathyroid lesions as well as diagnostic role. Although other study reported the sensitivity, specificity, diagnostic OR, and AUC of parafibromin IHC from meta-analysis, the result for the loss of parafibromin expression in various parathyroid lesions was not shown [28]. In addition, the correlation between parafibromin expression and survival was not shown in the previous meta-analysis [28].

A diagnostic test accuracy review to elucidate the diagnostic role of parafibromin IHC was performed. Regardless of cut-off value, the pooled sensitivity and specificity of parafibromin IHC was 0.53 (95% CI: 0.46–0.59) and 0.96 (95% CI: 0.95–0.97), respectively. In a previous study, the ranges of sensitivity and specificity of parafibromin IHC were respectively 0–72.7% and 89.5–100% [2]. In addition, the ranges of sensitivity and specificity of PGP9.5 IHC, positive marker for PC, was 33.3–63.6% and 85.0–100%, respectively [2]. Kumari et al. reported the effectiveness of combined IHC markers [2]. The sensitivity and specificity of combined parafibromin and PGP9.5 was 60.0 and 96.8%, respectively. According to the current diagnostic test accuracy review, in cutoff 0% subgroup, the pooled specificity was 0.95 (95% CI: 0.93–0.93). There was no significant difference of the specificity between various cut-off subgroups. Taken together, the cut-off value of loss of parafibromin IHC expression as low than 1% may be suitable for predicting of PC.

There are a number of limitations to the current study. First, in eligible studies, cases with normal parathyroid were only fifteen. The rate of loss of parafibromin IHC expression was 0.084 (95% CI: 0.017–0.328) in normal parathyroid (data not shown). The impact of intratumoral heterogeneity specimen type could be considered. However, there is limitation in interpretation due to small number of included cases. Second, eligible studies used various antibody clones, IHC methods, and cutoff from various populations. In addition, the heterogeneity between eligible studies may be present and the interpretation of the results performed through random-effect model. Third, on pathologic examination, only a portion of tumor could be histologically and immunohistochemically evaluated. Confirmation for complete loss of parafibromin IHC expression may be not easy before investigation of entire parathyroid lesion. In addition, the possibility of intratumoral heterogeneity of parafibromin IHC should be considered. If heterogeneity is present, this heterogeneity can affect on the rate of loss of parafibromin IHC expression.

In conclusion, loss of parafibromin IHC expression was significantly higher rate in PC than in other parathyroid lesion and showed higher diagnostic accuracy. Parafibromin IHC could be useful for diagnosis and differentiation of PC from other parathyroid lesion in daily practice. In addition, evaluation of parafibromin IHC might be helpful in predicting prognosis of PC.

## Abbreviations

APA: atypical parathyroid adenoma
AUC: area under the curve
HR: hazard ratio
IHC: immunohistochemistry
OR: odds ratio
PA: parathyroid adenoma
PC: parathyroid carcinoma
SROC: summary receiver operating characteristic
